# Activity Based Anorexia as an Animal Model for Anorexia Nervosa–A Systematic Review

**DOI:** 10.3389/fnut.2019.00069

**Published:** 2019-05-21

**Authors:** Martha A. Schalla, Andreas Stengel

**Affiliations:** ^1^Department for Psychosomatic Medicine, Charité Center for Internal Medicine and Dermatology, Charité-Universitätsmedizin Berlin, Corporate Member of Freie Universität Berlin, Berlin Institute of Health, Humboldt-Universität zu Berlin, Berlin, Germany; ^2^Department of Psychosomatic Medicine and Psychotherapy, Medical University Hospital Tübingen, Tübingen, Germany

**Keywords:** eating disorder, food restriction, hypophagia, hyperactivity, mice, rats, self-starvation, weight loss

## Abstract

Anorexia nervosa (AN) is a severe eating disorder affecting around 1 per 100 persons. However, the knowledge about its underlying pathophysiology is limited. To address the need for a better understanding of AN, an animal model was established early on in the late 1960's: the activity-based anorexia (ABA) model in which rats have access to a running wheel combined with restricted food access leading to self-starving/body weight loss and hyperactivity. Both symptoms, separately or combined, can also be found in patients with AN. The aim of this systematic review was to compile the current knowledge about this animal model as well as to address gaps in knowledge. Using the data bases of PubMed, Embase and Web of science 102 publications were identified meeting the search criteria. Here, we show that the ABA model mimics core features of human AN and has been characterized with regards to brain alterations, hormonal changes as well as adaptations of the immune system. Moreover, pharmacological interventions in ABA animals and new developments, such as a chronic adaptation of the ABA model, will be highlighted. The chronic model might be well suited to display AN characteristics but should be further characterized. Lastly, limitations of the model will be discussed.

## Introduction

Anorexia nervosa (AN) is characterized by underweight, self-induced weight loss achieved by food restriction or increased physical activity, endocrine alterations, and disturbance of body image affecting mostly young women with a 12-month prevalence of 0.4% among adolescents and young adults (1). The loss of body weight resulting in body mass index (BMI) values under 17.5 kg/m^2^ leads to various somatic symptoms affecting humoral and central nervous signaling as well as cardiovascular and gastrointestinal functions (2). To fulfill the diagnostic DSM-V criteria of anorexia nervosa a patient must show a restriction of energy intake inducing low body weight, a fear of gaining weight or behavior preventing weight gain as well as a disturbance of body image or lack of understanding of the danger of low body weight. Two subtypes of anorexia nervosa can be distinguished: restrictive AN characterized by restrictive calorie intake and binge-purging AN with self-induced vomiting (or other means to purge). It is to note that patients with AN often have psychiatric comorbidities such as affective, anxiety, obsessive-compulsive, and substance abuse disorders (3). These complications account for the high annual mortality rate of the disease, namely of 5.4 deaths per 1,000 person-years, representing the highest mortality rate among all psychiatric diseases (4).

Therapeutic interventions encompass nutritional and psychotherapy as well as—where applicable—drug therapy; however, the relapse rate is high ranging between 35 and 41% within 18 months (5). Consequently, there is a need for more efficient therapeutic options which requires better understanding of the underlying pathomechanisms responsible for the development of AN. To increase the knowledge about diseases, animal models depicting alterations of the disease can be very helpful.

In the late 1960's, core features of AN, namely hyperactivity, reduction of food intake and weight loss were observed in rats exposed to food restriction combined with access to a running wheel. Those animals increased their activity (6) and reduced food intake (7) leading to self-starvation and hyperactivity further aggravating weight loss. This negative relationship between food intake and activity seemed counterintuitive at first; however, subsequently it was hypothesized that an increase of activity allows animals/individuals to reach an area with stable food sources, thereby securing survival (8). Moreover, hyperactivity was also shown to suppress appetite (8) and to be an attempt to avoid a drop of body temperature (9). Additionally, in patients suffering from AN the drive for hyperactivity is positively correlated with anxiety, suggesting increasing activity as a mean to reduce anxiety (10).

Finally, the observation of self-starving in male rats which had unlimited access to a running wheel and restricted (1 h/day) access to food led to the establishment of ABA as a model of AN (11). Noteworthy, food-restricted male animals without a running wheel stabilized their body weight on a lower level, pointing toward the great importance of activity in this animal model (11). Consequently, the model was termed activity-based anorexia (ABA), sometimes also called exercise-induced anorexia or food restriction-induced hyperactivity (11). The standard ABA protocol consists of a 1–2 h feeding period during the light or dark phase, combined with a 22–24 h wheel access (11), leading to increasing hyperactivity and decreasing food intake over time. In contrast, when food access is not restricted in time but a fixed amount of food is given in the “semi-starvation-induced hyperactivity” model, no (further) reduction in food intake can be observed as indicated by similar intake of food in food restricted animals with and without access to a wheel (12). Thus, only ABA depicts a reduction of food intake/loss of appetite as observed in human AN. Most of the more recent ABA protocols include an acclimatization period to the wheel prior to the start of food restriction. The ABA protocol is usually pursued until a certain weight loss criterion, frequently defined between 15 and 30% weight loss (11), is reached. Additionally, a well-established variation of the ABA protocol is the gradual reduction of food access over several days (13). Most recently, several protocols describe prolonged endurance of food restriction and wheel exposure over a period of 55 days (14).

The present systematic review will present the current knowledge on the ABA model, discuss alterations induced by the model, highlight pharmacological options tested under these conditions and also mention limitations of the model. Lastly, gaps in knowledge will be addressed to stimulate further research.

## Methods

**Figure 1:**
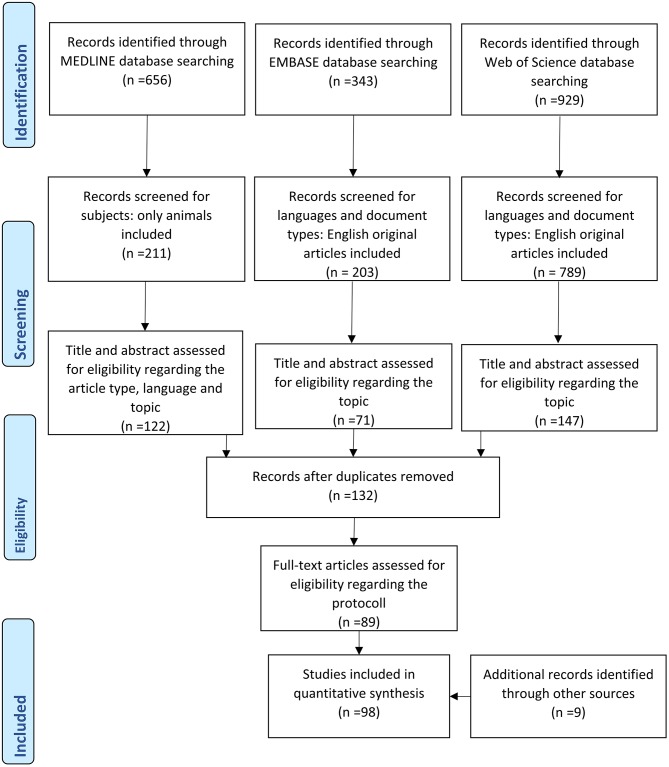
PRISMA flow chart.

For the systematic data search the PRISMA guidelines were applied. The data bases Pubmed—Medline and Web of Science were searched using the following search terms: “Activity based anorexia,” “Semi starvation induced hyperactivity,” “Exercise induced anorexia,” and “Food restriction induced hyperactivity.” Additionally, the EMBASE database was searched using the terms “Anorexia nervosa AND rat” and “Anorexia nervosa AND mouse.” The search was performed on October 20th 2018. Selection criteria applied were original publications, animal studies and English language. Thus, the exclusion criteria encompassed reviews, editorials, human studies, und manuscripts written in another language than English. In the manual screening all publications were identified investigating rodents exposed to food restriction in combination with running wheel access. Studies lacking one of these two characteristics were not included into the final list of publications. However, some are still mentioned in the manuscript to provide background information or are used for comparison. Several additional references were identified through review of the publication lists of the included articles for background information. Of those, nine were included into the final selection of references. After selection, 102 publications were included in this systematic review (Figure 1).

## Influencing Factors

Various versions of ABA protocols were used so far as presented in Table 1. Consequently, several influencing factors such as pre-exposure, ambient temperature and sound, handling and maternal separation, diet and food access, activity, sex, strain and genetics have been described able to affect food intake, activity and thus body weight loss during the development of ABA. These influencing factors along with the outcomes are summarized in detail in Table 2.

**Table 1A:** ABA protocols and respective outcomes (≥3 similar protocols of one research group).

**Species**	**Food**	**Wheel access**	**Acclimatization**	**Duration**	**Body weight loss**	**References**
Male C57Bl/6J mice	Progressive limited food	Free access	5 days of wheel	12 days	25%	(13)
	Access from 6 h/day (day 1) to		Acclimatization		23%	(15)
	3 h/day (day 4) at beginning of				26.7%	(16)
	Dark phase				22.5%	(17)
					22.7%	(18)
Female C57BL/6J mice					12%	(15)
					15%	(19)
				5 days and 12 days	12 and 15%	(20)
				5 days and 9 days and 12 days	12 and 16% and 14%	(21)
Female adolescent Sprague Dawley (SD) rats	1 h/day at the onset of dark phase	Free access	48 h of wheel acclimatization	3 days	16.7%	(22)
			3 days of wheel acclimatization	5 days	17.9%	(23–26)
				3 days	11.5%	(27)
				2–4 days	25%	(28)
			24 h of wheel acclimatization		20%	(29)
			4 days of wheel acclimatization	5 days	13.3%	(30)
				4 days	13.3%	(31)
Female C57Bl/6J mice	1 h/day at the onset of dark phase		2–3 days wheel acclimatization	2 days	22.9%	(32)
	2 h/day at the onset of dark phase		4 days of wheel acclimatization	3 days	22.2%	(33)
Male mice			5 days of wheel acclimatization	4 days	22.3%	(34)
Male and female C57Bl/6J mice					Male: 26.3% Female: 27%	(35)
Male adolescent SD rats	1.5 h/days	22.5 h	None	4 days	25 %	(36)
	1.5 h/day at the onset of light phase			3.1–3.2 days	26%	(37)
				3 days	26%	(38, 39)
				5 days	25%	(40)
				Not mentioned	25%	(41–43)
SD rats				male: 4 and 5 d, female: 6 and 7 days	25 and 30%	(44)
Female Long–Evans rats	2 h/day at the onset of dark phase	Free access	None	6 days	25%	(45)
				5 days	25.9%	(46)
	2 h/day during dark phase		10 days of wheel acclimatization	5 days	23%	(47)
Female SD rats	1.5 h/day at the onset of dark phase	Free access	10 days of wheel acclimatization	6 days	12%	(48)
Female adolescent SD rats				4 days	17.2%	(49)
	2 h/day at the onset of dark phase for 5 days, then 1 h/day at the onset of dark phase 1 h/day at the onset of dark phase		7 days of wheel acclimatization 10 days of wheel acclimatization	10 days	22.4% 29% lost 25% 86% lost 25%	(50)
SD rats	1.5 h food access during light phase	22.5 h	2h/2 days of wheel acclimatization	Male: 7 days; Female: 6 days	25%	(51)
Wistar rats				Male: 3 days; Female: 7 days	25%	(52)
Female SD rats			2 h/3 days of wheel acclimatization	4–7 days	20%	(53)
Female SD rats Male SD rats			2 h/day for 3 days of wheel acclimatization	5 days 4 days	21.3% 17.9%	(54)
Male albino Wistar rats	1.5 h during light phase	22.5 h	2 h/4 days of wheel acclimatization	7 days	25%	(55)
Male SD rats			2 h/3 days of wheel acclimatization	3–7 d (median 4 days)	25%	(56)
				5–9 days	20%	(57)
Female C57Bl/6J and Female DBA/2J mice	2 h/day at the onset of dark phase	Free access	3 days of wheel acclimatization	4 days	15% 22.5%	(58)
			7 days of wheel acclimatization	3 days	17.8% 10.5%	(59)
Female KK mice Female AKR mice m Female NZW mice Female A/J mice Female C3H mice Female C57BL mice Female WSB mice Female BALB mice Female CAST mice Female FVB mice Female DBA mice Female Wistar rats	5 pellets of about 1.2 g 2 h at the beginning of the dark phase 12 pellets of about 1.2 g 1.5 h at the beginning of the dark phase	None	7 days of wheel acclimatization 10 days of wheel acclimatization	4 days 5 days	100% lost 15% 100% lost 15% 100% lost 15% 88% lost 15% 85% lost 15% 80% lost 15% 30% lost 15% 28% lost 15% 25% lost 15% 15% lost 15% 0% lost 15% 0% lost 25%	(60)
Female Wistar rats	1 h/day at the onset of dark phase	Free access	10 days of wheel acclimatization	4 days	17.5%	(61)
			15 days of wheel acclimatization	6 days	24.2%	(9)
			10 days of wheel acclimatization	7 days	22.5%	(62)
	2 h/day and 1h/day			6 days	19.2 and 29.3%	(63)
	1 h/day at the onset of dark phase			7 days	25%	(64)
				5 days	18.7%	(65)
				7 days	18.3%	(66)
Female Balb/cJ mice	6 h/day from during light phase	Free access	9 days of wheel acclimatization	6 days	22.5%	(67)
				4 days	25%	(68)
Female Balb/cJ mice and Female A/J mice Female Balb/cJ mice	2 h/day during light phase 2 h 4 h 6 h 8 h 10 h		7 days of wheel acclimatization 9 days of wheel acclimatization	4 days 6 days 3 days 3 days 3 days 11days 11 days	30% 25% 27.5% 27.5% 25% 10% 5%	(69)
Female Wistar rats	1.5 h/day during light phase	Free access	None	7 days	15%	(70)
Male Wistar rats				9 days	20%	(71)
Wistar rats	1 h/day during light phase			5 days	15%	(72)
Male and female Wistar rats	1.5 h/day at the onset of light phase	22.5 h	Not mentioned	Male: 9 days; Female: 10 days	20%	(73)
Female SD rats	1.5 h during light phase	Free access	7 days of wheel acclimatization	14 days	22%	(74)
					23.0%	(75)
					25%	(76)
Female Wistar rats	1.5 h/day at the onset of dark phase	Free access	10 days of wheel acclimatization	6 days	20%	(77)
				4 days	21%	(78)
				6 days	17.3% 14.2%	(79)
	1 h/day at the onset of dark phase 1 h/day at various time points during dark phase			4 days	13.4% 17.4%	(80)
Female Wistar rats Female C57Bl/6 mice	1 h/day at the onset of dark phase 2 h/day at the onset of dark phase		10 days of wheel acclimatization 5 days of wheel acclimatization	5 days 3 days	25% 22%	(81)

**Table 1B:** ABA protocols and respective outcomes (≥3 similar protocols).

**Species**	**Food**	**Wheel access**	**Acclimatization**	**Duration**	**Body weight loss**	**References**
Female SD rats	1 h/day immediately prior to the dark phase	23 h	1.5 weeks of wheel acclimatization	5–18 days	20%	(82)
Female SD rats	1.5 h/day in middle of light phase	22.5 h	1 day of acclimatization to food restriction	10 days	18.9%	(83)
Male albino rats	1 h/day	22.5 h	1 day of acclimatization to food restriction	6 days	16%	(84)
Male SD rats	1 h/day during dark phase	23 h	3 days of acclimatization to food restriction	9 days	25%	(85)
Male and female rats	1.5 h/day	22.5 h	None	Male and Female: 4 days	25%	(86)
Female JCR:LA-cp rats	1.5 h/day at the onset of dark phase	22.5 h	10 days of acclimatization to food restriction	5 days	22%	(87)
Male JCR:LA-cp rats	1.5 h/day at the onset of dark phase	22.5 h	None	10 days	27%	(88)
Male Lewis ratsMale Brown-Norway ratsMale Fischer 344 rats	1.5 h/day during light phase	22.5 h	None	8 days 7 days 9 days	25.2% 24.7% 26.3%	(89)
Male SD rats	Food and water for 1.5 h/day during light phase	free access	None	4–14 days	12.1%	(90)
Male Wistar rats	1 h/day at the onset of dark phase	23 h	7 days of wheel acclimatization	7 days	25%	(91)
Female ICR mice	At the onset of dark phase	0.5 h/5 days /week	None	21 days	25.6%	(92)
Female SD rats	1.5 h/day at the onset of dark phase	free access	5 days of wheel acclimatization	5 days	22.5%	(93)
Female SD rats	1.5 h/day at the onset of dark phase	free access	11 days of wheel acclimatization	4–6 days	70% lost 20%	(94)
Female SD rats	1 h/day during dark phase	23 h	-	4–7 days	74%	(95)
Female Long-Evans rats	1 h/day during dark phase	Free access	7 days of wheel acclimatization 25 days of wheel acclimatization	3–6 days 5 days	20% 15%	(96)
Fem. adolescent Long-Evans rats	1 h/day before onset of dark phase	Free access	8 days of wheel acclimatization	4 days	40%	(97)
Male SD rats	90 min during light phase	22.5 h	7 days of acclimatization to food restriction	7 days	15.3%	(98)
Male C57/BL6J mice	3 h/day restriction proceeded as follows: 6 h/day on day 1.5 h/day on day 2, 4 h/day on day 3 and 3 h/day on day 4 progressive restrictive feeding schedule (6 h/day) decreasing by increments over a protracted period (21 days) to 2 h day	Free access	9 days of wheel acclimatization 7 days of wheel acclimatization 17 days of wheel acclimatization	7 days 18 days 40 days	30% 28%	(99)
Male SD rats	1 h/day	23 h	None	6 days	28%	(100)
SD rats	1 h/day 4 h within the light phase	Free access	7 days of wheel acclimatization	6 days	Male: 30%; Female: 20%	(101)
Female Wistar rats	1 h/day during light phase fixed time variable time point	23 h	2 days of wheel acclimatization	9 days	21% 28%	(102)
Male SD rats	1 h/day at the onset of dark phase	23 h	Pre-exposed to food restriction Pre-exposed to wheel non-exposed	2 days 8 days 10 days	1% 31.5% 27.3%	(103)
Female SD rats	1.5 h/day at the onset of dark phase	Free access	7 days of wheel acclimatization	6 days	21%	(104)
Male and female ICR/CD1 mice	Gradual food restriction in the form of 3–4 h/day during dark phase	Free access	7 days of wheel acclimatization	5 days	23% 60% resistant 40% ABA	(105)
Female SD rats	2 h/day at the end of light phase (30g)	Free access	10 days of wheel acclimatization	10 days	23%	(106)
Female Wistar rats	2 h/day during dark phase 3 h/day during dark phase 4 h/day during dark phase	Free access	3 days of wheel acclimatization	21 days	18.5% (day 8) 5/10 died 11.1% weight gain (day 21) No weight loss No weight loss	(107)
Male Wistar rats	Increasing amounts of food (6–10 g) to keep weight loss between 5 g and 8 g/day during dark phase	Free access	None	10 days	31%	(108)
Male SD rats	3 h feeding period	Free access	7 of day of wheel acclimatization	7 days	12.5%	(109)

**Table 2:** Factors influencing ABA outcome.

**Influencing factor**	**Effects on ABA outcome**
Pre-exposure to: Restricted feeding	↑ Survival rate of 75% (110) Diminished food intake reduction, hyperactivity and body weight loss (103)
Feeding schedule	↓ Body weight loss (111)
Food restriction-induced weight reduction Low initial body weight Running wheel	↓ Food intake reduction, hyperactivity and body weight loss (103, 111) No effect on ABA development (98) No effect on ABA development (112) ↑ ABA vulnerability (113) Deaccelerated self-starvation, no effect on percentage of survival (110) ↑ Body weight loss and hyperactivity, food intake reduction (111) ↑ Body weight loss and hyperactivity, food intake reduction (103)
High ambient temperature Access to a warm plate	Deaccelerated body weight loss in male rats (55) ↓ Hyperactivity and body weight loss in male rats (56) ↓ Hyperactivity and body weight loss, food intake reduction (57) Reversed hyperactivity, preserved food intake in female rats (53) ↓ Hyperactivity and body weight loss in female rats (9)
Sound attenuation condition	Extended ABA duration, ↓ hyperactivity (54)
Daily handling Maternal separation of 180 min daily for 20 days postnatally Maternal separation for 180 min/day for 14 days	↓ Body weight loss (52) Delayed reaching the removal criterion of 20% weight loss (52) ↑ ABA resistance (greater survival) (51) Accelerated weight loss, ↑ activity, ↓ food intake in females (114). Prevented ABA in males (115).
Food presentation at irregular times Different food access durations Time of food presentation Food type Drinking	↑ body weight loss and hyperactivity, food intake reduction (102) 6 h/day: ↓ survival (69) 3 or 4 h/day: cessation of estrous cycle, body weight loss, food intake suppression and hyperactivity (107) 2 h/day: severe gastric lesions, ↑ mortality (107). At the onset of dark phase: prevented body weight loss (111). High fat chow/vegetable fat: prevented reaching of removal criterion (85) 0.88 M sucrose: prevented hyperactivity and body weight loss (116) Palatable food (2 h/day): binge eating (22). Wet mash/adaptation to drinking schedule: prevented ABA (117)
Running wheel access Pre-prandial/ food-anticipatory activity Postprandial activity	Wheel inaccessibility 4 h before feeding: diminished body weight loss (111) Induced sickness (118) Activity levels before ABA induction strongly predicted outcome of ABA (60, 101) ↓ Body weight loss (119) ↑ Body weight loss (119)
Female sex Male sex	↑ Food intake, hyperactivity, deaccelerated body weight loss (44) ↑ Body weight loss and hyperactivity, no ↑ vulnerability to ABA (120) ↑ Body weight loss and hyperactivity (121) ↓ Body weight loss and food intake reduction, ↑ hyperactivity during food intake period and post-prandial hyperactivity (13, 15) ↓ Hyperactivtiy during food intake period (35). ↑ Food intake reduction, hyperactivity, accelerated body weight loss (44) Body weight loss correlated with ↑ running (120) ↑ Mortality rate (20% weight loss in 3 days), body weight loss and food intake reduction, ↑ food-anticipatory/pre-prandial activity (13, 15) ↓ Food-anticipatory/pre-prandial activity (35)
Strain Genetics	C57BL/6J mice: ↓ hyperactivity, DBA/2J mice: ↑ hyperactivity (58) Brown Norway and Lewis rats: ↓ thymus weight (89) A/J mice: longer survival (69). Chromosome substitution strains 4, 12, 13: ↑ hyperactivity during the light phase hours/food restriction phase (122) Lean-prone rats: ↑ hyperactivity and accelerated body weight loss (87) Leptin receptor deficiency: prevented reaching weight loss criterion (87) α4βδ-GABAAR KO female rats: ↑ body weight loss and hyperactivity, food intake reduction (35) Reduced miR-340 expression: ABA resistance (105)

*↑, increase; ↓, decrease*.

The following main conclusions can be drawn: Maternal separation and daily handling should be omitted or be similar for all animals, since it significantly influences weight loss (51, 52). ABA occurs because wheel running interferes with adaptation to the feeding schedule, which is omitted when the feeding schedule is introduced before the running wheel. Warm ambient temperature could have an ameliorating effect on AN; thus, a standard ambient temperature should be used in ABA to reach the weight loss criterion. A feeding schedule for < 2 h in rats with standard chow and *ad libitum* access to water is sufficient to induce hyperactivity and weight loss, both indicators of ABA. It can be assumed that it is useful to study activity levels during the wheel adaption period in order to early on detect low susceptibility to ABA (60, 101). Obviously, wheel access is a crucial characteristic of ABA. Since pre-prandial activity increases weight loss (119), food-anticipatory activity seems to be an important factor for weight loss as well. Although in humans AN is more prevalent in females, early studies often used adult male rats to avoid the influence of hormones associated with both development and reproduction. However, in rats studies focusing on the effect of sex on ABA outcome showed that ABA seems to develop more effectively and rapidly in males; noteworthy, also food-anticipatory activity was more pronounced in males (13, 15). Since females take longer to reach the weight loss criterion (44), in order to mimic and better understand human AN, female animals should be examined in a more long term protocol. Comparing different ages, younger rats develop ABA more rapidly (123). To develop ABA in an adult rodent population takes substantially longer than in adolescence, probably resulting from the fact that—besides absence of significant body weight gain during this period—escalation of running is often blunted in adults compared to adolescent rats. As a consequence, adult animals can often be maintained in the paradigm for up to several weeks without reaching the maximum weight loss criterion. However, since younger animals are more vulnerable to ABA, young animals typically cannot be maintained in the paradigm for more than several days regardless of sex. Regarding the investigation of different strains, C57BL/6J mice might not be the first choice to examine effects of ABA (58), and A/J mice might be more useful in long-term protocols (69). Similar strain comparisons should be performed in rats.

## Effects of ABA

### Alterations of Hypothalamic Nuclei and Transmitters

One of the main roles of the hypothalamus is to regulate hunger, satiety, energy metabolism and ultimately body weight (124). Areas of the hypothalamus involved in food and water intake regulation encompass the arcuate nucleus [ARC, (125)], the dorsomedial hypothalamus (126), lateral hypothalamus [LHA (128)] the paraventricular nucleus [PVN (129)] and ventral medial hypothalamus (130); thus, the effects of ABA on the activity of the hypothalamus and its transmitters were studied extensively.

ABA in female rats activated neurons in the supraoptic nucleus [expressing oxytocin, related—among others—to anxiety (131)] and ARC [participates in the regulation of food intake as mentioned above (125)] compared to *ad libitum* fed rats as assessed using the neuronal marker c-Fos when perfused directly after the feeding period (74). Food-anticipatory activity in the running wheel correlated with c-Fos expression in the dorsomedial hypothalamus [DMH, involved in the regulation of food and water intake as well as body weight (126)] in female ABA Wistar rats (80). Interestingly, ABA rats on a random feeding schedule, which did not develop food-anticipatory behavior, displayed a negative correlation between neuronal activity in the ARC and body weight loss (80). Before food access and during pre-prandial activity, c-Fos expression and ARC, PVN as well as in the nucleus accumbens [NAc, involved in the processing of motivation, aversion and reward, lesions result in increased food intake and weight gain; (127)] was observed to be reduced in male rats undergoing ABA when provided access to 0.88 M sucrose, but not after 0.002 M saccharin in a comparable sweetness, giving rise to an attenuating effect of sucrose on wheel running possibly induced by inhibition of hypothalamic activation through corticosterone (116).

Lateral hypothalamus electrical stimulation (100 Hz and 25, 50, and 75% of the maximal stimulation amplitude) on consecutive days during four test sessions significantly decreased locomotor activity in female ABA rats with no effect on food intake or survival time (72). Future studies should focus on other brain areas targeted by electrical stimulation to alter features of AN.

In female mice ABA induced an increase of hypothalamic protein synthesis. These changes of the hypothalamic proteome especially affected proteins involved in glycolysis or in citric acid cycle (21), giving rise to an adaptively enhanced energy metabolism. Additionally, proteins limiting oxidative stress were altered (21). DNM1L, implicated in mitochondrial fission, was overexpressed under conditions of ABA leading to an increased number of mitochondria, while increased dynamin-1 and LC3II/LC3I ratio indicated an activation of autophagy (21). Taken together, ABA leads to an adaptation of the hypothalamic proteome inducing autophagy and mitochondrial changes.

#### Pro-Opiomelanocortin (POMC), Cocaine- and Amphetamine-Regulated Transcript (CART), Agouti-Related Protein (AgRP), and Neuropeptide Y (NPY)

The protein POMC, cleaved into various active peptides such as melanocyte-stimulating hormones, is essential in the regulation of food intake inducing satiety; thus, its absence can lead to obesity (132). Among others, POMC is expressed in the hypothalamus (133). In female ABA rats, arcuate expression of POMC protein was reduced 2-fold (61). In contrast, another study reported a transient up-regulation of POMC mRNA levels in the ARC, pointing toward increased anorexigenic signaling in female ABA rats (64). This discrepancy might be due to dynamics of peptide expression in the course of ABA development. Similarly, food restriction 1 h food access per day at the beginning of the dark phase), regardless of the level of physical activity, elevated melanocortin receptor (involved in POMC signaling) expression in the ventral medial hypothalamus (134). This is likely of physiological importance as intracerebroventricular (icv) infusion of AgRP, an orexigenic peptide expressed in the ARC with inverse agonistic activity on melanocortin 3 and 4 receptors (135), increased the survival rate by decreasing physical hyperactivity and blunting suppression of food intake (134). Conversely, chronic administration of alpha-melanocyte stimulating hormone (α-MSH), an anorexigenic cleavage product of POMC (136), into the lateral ventricle stimulating the melanocortin receptors and the hypothalamus-pituitary-adrenal (HPA) axis [mediating the response to stress, regulating digestion, and energy expenditure as well as inhibiting reproductive functions (137)] elevated running wheel activity and reduced food intake in rats resulting in higher vulnerability to develop ABA (63). Arcuate protein expression levels of AgRP and NPY [orexigenic hypothalamic neuropeptide (138)] were elevated 5-fold and correlated negatively with relative body weight and white adipose tissue mass (61). A recent study corroborated these data by showing that ABA in female Sprague Dawley rats led to higher NPY, AgRP but also POMC expression (48). Additionally, increased levels of NPY mRNA in the ARC were observed in female ABA rats compared to those having *ad libitum* access to food (139). In female rats exposed to a feeding time of 2 h/day; daily icv infusion of NPY at the end of the light phase over a period of 7 days accelerated development of ABA by reducing food intake and increasing wheel running (139). Combination of food restriction and enhanced physical activity in male C57BL/6J mice elevated hypothalamic protein expression of AgRP, while food restriction alone increased NPY and AgRP, indicating that anorexic-like conditions disrupt hypothalamic circuitries (140). Therefore, it can be hypothesized that ABA induces alterations of neuropeptides including AgRP, NPY, POMC, and CART in first order neurons (in e.g., ARC, LHA) able to negatively impact on energy balance.

#### Endorphins

The peptide β-endorphin is also derived from POMC and mainly expressed in the hypothalamus. Dynorphin-A is another endorphin with opioid-like effects. In male rats voluntary exercise under food-restricted conditions elevated β-endorphin in the ARC and dynorphin-A in the supraoptic hypothalamic nucleus following 2-deoxy-D-glucose (2DG) stimulation (38). In addition, in the suprachiasmatic nucleus dynorphin-A was increased (37). Female rats undergoing the ABA paradigm did not display alterations of hypothalamic β-endorphin concentration compared to controls (141). These observations in ABA resemble data of increased opioid activity in humans with AN (142); thus, further studies on the role of opioid signaling in AN are warranted.

#### Central Orexin and Leptin

Female rats with a proactive stress-coping style to prenatal stress, assessed by the defensive burying test, displayed elevated hypothalamic mRNA orexin expression, higher levels of DNA methylation of the orexin gene as well as reduced leptin levels while their ghrelin levels were increased during ABA (48). In male ABA rats, chronic leptin infusion using minipumps, concomitantly to the initiation of food restriction for 7 days, suppressed the 300% increase of baseline physical activity observed in restrictively fed vehicle animals (12). Also, chronic subcutaneous (sc) application of leptin after initiation (6 days) of semi-starvation-induced hyperactivity abolished this alteration (12). Chronic leptin treatment (icv via osmotic minipump, 4 μg/d) in female ABA rats decreased running wheel activity, food intake, and increased energy expenditure by thermogenesis (65). Similarly, in female rats undergoing ABA, acute central leptin injections into the lateral ventricle and microinjection of leptin into the ventral tegmental area at day 4 diminished running wheel activity (79), pointing toward a role of decreased leptin signaling in hyperactivity.

#### Corticotropin-Releasing Factor (CRF)

CRF, expressed in the PVN and stimulating adrenocorticotropic hormone (ACTH) secretion, is a hallmark regulator of the HPA axis and thus involved not only in the stress response but also energy metabolism (137). Seven-day running wheel access increased CRF mRNA expression in the DMH, but not in the PVN, of male rats (143). Similarly, ABA in female Sprague Dawley rats did not alter CRF mRNA concentrations in the PVN (86). Interestingly, female rats that developed ABA during adolescence presented increased anxiety-like behavior associated with increased expression levels of CRF mRNA in the PVN and the central nucleus of the amygdala in adulthood, whereas food restriction alone did not induce these changes (144). Another study showed that ABA elevated c-Fos in CRF positive neurons of the PVN and DMH (76). The effects of wheel running on meal size could be reversed by icv injection of the CRF antagonist α-helical-CRF_(9−41)_, additionally increasing DMH CRF mRNA expression (143), pointing toward a crucial role of CRF in the development of ABA. In another study, icv injection of the competitive CRF antagonist SHU9119 had no ameliorating effect on ABA, while treatment with the inverse agonist AgRP_(83−132)_ did (64). Therefore, the role of CRF in the development and maintenance of ABA needs further investigation.

#### Nesfatin-1

Nesfatin-1 is an anorexigenic neuropeptide found predominantly in the hypothalamus (and peripherally in the gastric mucosa) also involved in the modulation of gastrointestinal functions (145). Phenotyping studies in female rats showed that following a 7-day wheel acclimatization +14 day food restriction protocol with or without a running wheel, the number of activated nesfatin-1 immunoreactive cells was increased in the PVN, ARC, DMH, dorsal raphe nucleus and the rostral raphe pallidus compared to *ad libitum* fed and activity (running wheel) controls (75). Also, food restricted rats showed a trend toward an increase of NUCB2/nesfatin-1 (most antibodies do not distinguish between full length NUCB2 and nesfatin-1) protein expression in the PVN, ARC, LC, and DMH, while in rats with access to the running wheel only no altered expression of nesfatin-1 was observed compared to *ad libitum* fed control rats (75). The differences between ABA and food restricted rats indicate central alterations independent of a simple body weight reduction. Taken together, alterations in expression patterns can be observed in motor and higher food intake circuitries due to ABA, likely underlying/contributing to the effects on food intake and locomotor activity. These activity patterns resemble those observed in humans with AN, e.g., altered cortical processing of high and low-calorie food pictures compared to healthy controls has been described (146).

#### Serotonin

Serotonin (5-HT) as one of the most important central neurotransmitters is widely expressed in the human brain. It is thus involved in various regulatory processes, modulating—among others—mood, anxiety, aggression, and hunger (147). The serotonin receptors can be subdivided into seven subtypes: 5-HT_1_-5-HT_7_, all of which are G-protein coupled receptors with the exception of 5-HT_3_, which is an ion channel. Serotonin's appetite-suppressing effect is mainly mediated by 5-HT_1B_, 5-HT_2C_ and 5-HT_6_ receptors in the ARC and PVN (148). In humans, evidence showing a robust effect of antidepressants on body weight gain in AN is limited so far (149). However, the combination of selective serotonin re-uptake inhibitors (SSRI) with psychotherapy can exert positive effects on anxiety, depression, and compulsive thoughts (150).

Dietary restriction inducing hyperactivity reduced central, especially hypothalamic, serotonin signaling (151). A reduction of serotonin concentration has also been observed in AN patients (152). Daily administration of fluvoxamine (7 d, orally), a SSRI, suppressed the increase in running activity after feeding without affecting body weight loss or food intake in ABA rats (109). In female ABA mice, chronic oral treatment with fluoxetine, another SSRI, elevated food intake, and reduced activity without any effects on survival (69). Similarly, female ABA rats, treated ip with fluoxetine for 5 weeks, showed an increased food intake, displayed decreased running activity and lost less weight compared to saline treated rats (83).

Agonists with high affinity for the 5-HT_1C_ receptor [located in cortical and subcortical neurons of the hippocampus, thalamus, and monoaminergic cell groups (153)] blocked semi-starvation-induced increased running wheel activity in rats (108). Daily sc injections of 8-OH-DPAT, an agonist of the 5-HT_1A_ receptor (found pre- and postsynaptically, ubiquitously located, and stimulating cAMP formation in the respective neurons), administered 40 min before feeding for 10 days prevented hyperactivity, subsequently reducing body weight loss in female ABA rats (46). In contrast, hyperactivity, induced by restricted access to food in female rats, was enhanced by acute sc injection of 8-OH-DPAT (151), pointing toward different effects of 5-HT_1A_ and 5-HT_7_ activation in ABA and food-restricted animals.

Fenfluramine (continuously infused sc during 1 week), an appetite suppressant activating 5-HT_2C_ receptors and releasing serotonin resulting in increased extracellular serotonin levels, did not affect food intake, wheel running, body weight, hypothermia or HPA axis activity, while inducing hypodipsia, elevated plasma osmolality, and arginine-vasopressin expression levels in the hypothalamus (62). In another study, ABA rats chronically sc infused with fenfluramine using mini-pumps had a greater susceptibility to ABA due to a reduction of food intake (43). Similarly, fenfluramine (acute ip injection) administered 1.5 h prior to the daily 2-h period of food access in female ABA rats induced an accelerated weight loss (45). Female ABA rats treated with parachlorophenylalanine, an irreversible tryptophan hydroxylase inhibitor depleting serotonin, showed decreased food intake and increased running activity resulting in increased body weight loss (83), suggesting an inverse correlation between central serotonergic activity and vulnerability to develop ABA. Taken together, these observations indicate that signs of ABA (weight loss, hyperactivity) can be intensified by increasing peripheral 5-HT signaling, while centrally a reduction exerted a similar effect.

Fluoxetine, another SSRI, was shown to exert various effects on ABA animals. In detail, it decreased dynorphin-A content under ABA conditions (37), induced a pathological elevation of vasopressin in the suprachiasmatic hypothalamus, while a physiological increase in plasma vasopressin was observed (37, 42). Additionally, it reduced oxytocin secretion in ABA animals, while increasing oxytocin in control animals (37, 42). Lastly, fluoxetine had no effect on parameters like blood glucose, plasma insulin or insulin-like growth factor 2 (IGF-2) concentrations in ABA (154).

### Alterations of Central Structures and Transmitters Involved in Reward-Motivated Learning

Eating behavior is strongly linked with the reward system. Also, eating disorders may be interpreted as reward-dependent since e.g., reducing eating is perceived as rewarding in AN (155). Thus, various studies examined central areas involved in reward in the context of ABA.

Examining brain activity using a micro PET-CT, increased cerebral 18F-fluorodeoxyglucose uptake was observed in male ABA rats in the mediodorsal thalamus [playing a role in memory and cognition (156) and body weight regulation (157)], ventral pontine nuclei and cerebellum [modulates motor activity, involved in classical conditioning (158)] compared to active and inactive *ad libitum* fed controls (71). On the other hand, ABA was associated with a reduced uptake in the left rhinal and bilateral insular cortex [responsible for gustatory and viscerosensory processing; (127)] as well as bilateral ventral striatum [involved in processing of motivation, aversion and reward, lesions results in increased food intake and weight gain; (127)] compared to control animals without food restriction and without access to running wheels (71). Noteworthy, brain metabolism in the cingulate [processing reward by linking actions and emotions resulting in learning (159)] and surrounding motor and somatosensory cortex was positively correlated with body weight (71). These data suggest a significant role of central structure involved in reward signaling in the development and/or maintenance of ABA.

Similarly, electrical stimulation or an electrolytic lesion of the mediodorsal thalamus did not affect signs of ABA in female rats that already developed ABA, while a preventive lesion selectively decreased hyperactivity in ABA later on (70). Future studies using electrical stimulation should include additional brain areas to possibly affect features of AN.

#### Dopamine

Dopamine expressed in the ventral tegmental area (VTA) as part of the mesocorticolimbic dopamine system is a key regulator of the reward and motivation system (160). The dopamine receptors are categorized into two families; the D_1_ and D_5_ receptors belong to the D_1_-like family, which are G-protein coupled, increase cAMP and can be mainly found post-synaptically, while D_2_, D_3_, and D_4_ receptors represent the D_2_-like family inhibiting cAMP formation and are located both pre- and post-synaptically. In brief, stimulation of D_1_ receptors results in dilatation of cerebral vessels. D_2_ receptors modulate motor activity similar to D_4_ receptors, while D_3_-receptors, located in the limbic system and cortex, are involved in cognition (161).

Restricted food access elevated mRNA expression levels of neuronal cell adhesion molecule 1 (NCAM1), involved in the formation and modulation of synaptic contacts in the VTA of Balb/cJ female mice (67).Wheel running alone elevated mRNA expression of the growth factor, brain-derived neutrotrophic (BDNF) in the VTA. When both conditions were combined, no effects on BDNF or NCAM1 mRNA expression within the mesocorticolimbic pathway could be observed (67). Female A/J inbred mice with typical signs of ABA showed elevated dopamine D_2_ receptor expression in the caudate putamen (59). In female ABA rats, dopamine release in the nucleus accumbens was found not to be elevated during the initiation of food-anticipatory behavior but it was increased during food intake; additionally, serotonin levels were decreased and circadian activity was diminished (78). An elevation of dopamine levels has also been observed in AN patients (162).

As mentioned above, an effect of antidepressants on significant body weight gain in patients with AN could not be detected so far (149). Among psychotropic drugs, olanzapine, a non-selective modulator of various neurotransmitter systems such as dopamine signaling, was the only medication found to accelerate weight gain and reduce mealtime anxiety in AN; however, inconsistent data exist (150). In an ABA model, during food-anticipatory activity (locomotor activity that occurrs 2 h before the availability of food), levels of dopamine and its metabolites in the striatum and midbrain were upregulated (163). Consequently, treatment of ABA BALB/cJ mice with olanzapine (orally applied daily for 7 days) antagonizing dopamine and serotonin receptors increased survival by decreasing food-anticipatory activity (164). Similarly, ABA rats treated with olanzapine (sc daily for 7 days using an osmotic minipump) displayed a decreased running wheel activity rate, starvation-induced hypothermia and activation of the HPA axis, indicated by decreased levels of ACTH, corticosterone and adrenal weights without affecting food intake, while body weight loss was decreased and ABA development diminished (66). Daily oral treatment with the D_2/3_ receptor antagonists eticlopride and amisulpride in ABA Balb/cJ female mice decreased weight loss and hypophagia, resulting in increased survival (68). Additionally, amisulpride reduced weight loss and hypophagia to a higher extent compared to olanzapine (68). Similarly, the D_3_ receptor antagonist SB277011A or the D_2_ receptor antagonist L-741,626 elevated survival (68). Also, application of chlorpromazine, another dopamine receptor antagonist, reduced hyperactivity, leading to a 75% decreased mortality (11). In male rats, chlorpromazine (intraperitoneally, ip) blocked food suppression after wheel running during the light phase in an acute model of ABA (165). ABA rats treated chronically with the non-selective dopaminergic D_1_ and D_2_ antagonist cis-flupentixol reduced body weight loss and increased food intake (77). The D_1_ receptor antagonist SCH23390 as well as the D_2_ receptor antagonist raclopride (acute ip injection) significantly decreased food-anticipatory activity compared to controls, while co-administration of both showed an additional effect on the reduction of food-anticipatory activity (163). Noteworthy, dopamine D_1_-like antagonists such as SCH23390 did not alter survival (68). The hypothesis that antagonizing dopamine signaling has beneficial effects by increasing food intake and body weight and reducing hyperactivity in ABA should be substantiated in future studies.

Excitation of the reward pathway by means of a dual viral strategy involving retrograde transport of Cre to the ventral tegmental area and coincident injection of DREADD receptors inducing recruitment of a large proportion of VTA-NAc dopaminergic projections decelerated establishment of ABA in female rats by increasing food intake, food-anticipatory activity and reducing weight loss (93). In female rats a direct correlation between the intensity of activity and the severity of withdrawal symptoms, assessed following sc naloxone injection, was observed; thus, ABA rats displayed the most withdrawal symptoms compared to restrictively fed, *ad libitum* fed and active controls (96). Taken together, these results indicate an alteration of the reward system during ABA in line with abnormal reward circuitries described in AN (166). Options to modulate the reward system modulating the severity of ABA should be examined further.

### Alterations of Hippocampal Structures and Transmitters

The hippocampus is responsible for transmitting memory content from short to long-term memory (167). Restricted feeding for 14 days increased the transcripts of the growth factor BDNF in the hippocampus of Balb/cJ female mice (67), suggesting a putative role of the hippocampus in ABA. In contrast, female A/J inbred mice undergoing the standard ABA protocol showed decreased BDNF expression in the hippocampus (59). Similar to these inconsistent data of BDNF alterations in ABA, studies on BDNF levels in AN have shown variable results (168).

In adolescent female Sprague Dawley rats, cell proliferation in the dentate gyrus and hilus region, but not in the subgranular zone and in the surrounding dorsal hippocampus and in the corpus callosum, was reduced after 3 days of ABA with a positive correlation between cell proliferation and body weight/food intake (29), indicating an effect of ABA in adolescents rather on gliogenesis than on neurogenesis. Female ABA rats showed reduced total dendritic length and dendritic branches in the stratum radiatum of the dorsal hippocampus, responsible for spatial learning and cognition; in contrast, branching in stratum radiatum of the ventral hippocampus mediating anxiety was elevated in ABA (24). Exercise mainly affected stratum radiatum, while food restriction influenced the stratum lacunosum moleculare in the dorsal and ventral regions (24), pointing toward pathway-specific alterations in the hippocampus due to ABA. ABA in female adolescent Sprague Dawley rats increased branching of ventral hippocampal pyramidal cells, while the same protocol in adulthood decreased branching of ventral hippocampal pyramidal cells without any effects on dendritic branching (25). The proportion of mature spines on dendrites was also altered due to ABA: in adolescent female ABA animals it resembled adult control animals since control animals doubled branching from adolescence to adulthood (25). The results underline the age-dependent vulnerability of hippocampus plasticity to ABA. Noteworthy, relapse of ABA decreased branching (25). Thus, the hippocampus is an important structure implicated in the development of ABA.

#### Hippocampal Gamma-Aminobutyric Acid (GABA)

In the hippocampus of female ABA rats a 6-fold increase of α4 subunits of α4βδ GABA receptors and a 130% increase of δ subunits of α4βδ GABA receptors, sufficient to increase tonic hippocampal inhibition, was observed compared to age-matched control females (23). GABAergic inhibition in the hippocampus strongly induces anxiety and additionally regulates plasticity (169). Similarly to the findings in the hippocampus, in the amygdala of female pubertal rats under ABA conditions an increase of membranous α4 subunits near excitatory synapses on dendritic shafts in the caudal basal amygdala accompanied by intracellular elevation of α4 subunits was observed, indicating a disinhibition of the excitability of the amygdala (26). Hyperactivity during food restriction in ABA adolescent female rats negatively correlated with α4βδ GABA receptor levels visible within 2 days of food restriction (27), suggesting a protection against ABA by inhibition of α4βδ-GABAARs in spines of CA1 pyramidal neurons suppressing physical activity.

A negative correlation was described between α4 subunit concentration at spines of pyramidal cells of the hippocampal CA1 with severity of ABA, measured as food restriction-elicited running activity during ABA (170), suggesting a protective role of α4 subunits counterbalancing the ABA-induced excitability of CA1 pyramidal neurons.

Contact lengths of axo-somatic contacts made by GABAergic axon terminals onto layer 5 pyramidal neurons were increased by 40% in female ABA mice; thus, the proportion of L5P perikaryal plasma membrane contacted by GABAergic terminals was elevated accordingly (171). Additionally, in female ABA mice a negative correlation was observed between contact length in the anterior cingulate cortex and overall wheel activity after food restriction and between contact length in the prelimbic cortex and wheel running especially during food availability in the restriction phase (171). Adolescent female C57BL/6 mice that developed ABA with food access during the first 2 h of the dark cycle all survived; when re-exposed to the same conditions after recovery for 7–11 days only some were vulnerable to ABA with those being vulnerable displaying a reduced GABAergic innervation on cell bodies and dendrites in CA1 pyramidal cells compared to resilient mice (32). In summary, this underlines that GABAergic innervation of hippocampal structures contributes to the protection of animals against ABA.

In rats, chlordiazepoxide (acute ip injection), a benzodiazepine, suppressed the decrease of food intake under conditions of ABA (172).

#### Hippocampal N-Methyl-D-Aspartat (NMDA)

While GABA is the main inhibitory, glutamate is the major excitatory neurotransmitter in the brain (173). Hippocampal NMDA, as part of glutamate receptors and ion channel protein, is involved in modulating learning, memory processing, and feeding behavior (174). Using electron microscopy hippocampal synaptic NR2A-NMDA and NR2B-NMDA receptor levels were observed to be increased in female ABA rats (31). In those animals, ABA severity positively correlated with synaptic NR2B-NMDA receptor levels (31). In rodents resilient to ABA that did not develop hyperactivity, reserve pools of NR2A-NMDA receptors in spine cytoplasm correlated with the suppressed physical activity (31). NR2A- and NR2B-NMDA receptors were related to spinous prevalence of an F-actin binding protein, drebrin, responsible for receptor insertion to and retention from synaptic membranes (31), indicating that increased NMDA receptor expression elevates the vulnerability to ABA. Noteworthy, anti-NMDA receptor encephalitis in humans, resulting in decreased receptor density, is also associated with abnormal eating behavior (175).

Subchronic treatment with agmatine (ip, for 10 days), an endogenous ligand of imidazoline and α2-adrenergic receptors that additionally selectively blocks the NMDA subclass of glutamate receptor channels, reduced hyperactivity, increased food intake and normalized body weight of female ABA rats, also decreasing corticosterone levels (106), probably resulting from restored body weight and increased food intake.

Clonidine, an α2-adrenergic receptor agonist, inducing sympatholytic effects such as a reduction of blood pressure (176) via negative feedback mechanisms was also tested under conditions of ABA. Chronic infusion of clonidine into the PVN of male ABA rats resulted in a dose-related increase in the susceptibility to ABA and a decrease in food intake; similarly, in heavy animals an increased susceptibility to ABA was observed after chronic infusion of clonidine into the PVN but without effect on food intake or wheel activity (41). Male rats receiving a continuous sc infusion of clonidine using osmotic minipumps and exposed to ABA showed an increase of food intake at a lower dose of clonidine and a stimulation of wheel activity at a higher dose, with no effects on weight loss (40), indicating that centrally applied clonidine increases the vulnerability to ABA, an effect mimicked by higher peripheral doses presumably crossing the blood-brain barrier.

#### Cannabinoids

A rich hippocampal expression of the type 1 cannabinoid (CB_1_) receptor suggests an important role of cannabinoids in the hippocampal network and memory formation (177).

ABA conditions increased absolute CB_1_ receptor binding using (18)F-MK-9470 in all cortical and subcortical brain areas in both sexes, which decreased again in the recovery phase (73). Elevation of relative CB_1_ receptor binding was observed in the hippocampus, inferior colliculus and entorhinal cortex in female ABA rats, which also normalized with weight regain in the recovery phase (73), giving rise to impaired endocannabinoid transmission under conditions of ABA, a finding also reported in humans with AN (178).

In male C57/BL6 mice undergoing ABA daily ip application of the phytocannabinoid delta(9)-THC 30 min before the dark phase reduced survival but increased feeding in the animals which did survive, while the anandamide analog OMDM-2 stimulated food intake without sufficiently reversing weight loss (99). Subchronic ip THC treatment 30 min before the onset of the dark phase in female ABA rats transiently increased food intake and also affected running wheel activity (179). The higher dose also decreased body weight loss accompanied by reduced energy expenditure and lipolysis (179). When combined with high fat diet, THC had the same effects but to a greater extent (179). Daily ip injection for 6 days with the CB_1_/CB_2_ receptor agonist Δ9-tetrahydrocannabinol or the CB_1_/CB_2_ receptor agonist CP-55,940 decreased body weight loss, physical activity, and plasma corticosterone levels while increasing leptin signaling in female ABA rats (104). Noteworthy, treatment was initiated at the start of a second ABA protocol after rats already experienced one ABA and one recovery phase (104). Overall, the cannabinoid system is able to increase food intake also under conditions of ABA. Noteworthy, although the CB_1_ receptor is highly expressed in the hippocampus and might mediate the effects mentioned above via the hippocampus, it should be kept in mind that this receptor is also found in the VTA and hypothalamus. It cannot be excluded that the orexigenic effects observed after CB_1_ receptor agonist application are mediated via VTA and hypothalamus, a hypothesis which should be examined further.

Consequently, male rats that orally received the CB_1_ receptor antagonist SR141716 over a period of 32 days prior to ABA starved faster, lost weight faster and increased the wheel running rate more rapidly compared to those without drug treatment (88). Rats with the same drug treatment but lacking a functional leptin receptor did not reach the starvation criterion of 25% body weight loss (88). Additionally, they displayed reduced wheel running as well as decreased levels of serotonin and its metabolic products in the hypothalamus and neural-reward areas including the nucleus accumbens compared to animals with the same dysfunctionality but without drug administration, suggesting an interaction between CB_1_ and leptin receptor signaling also implicated in regulating energy balance (88).

### Alterations in Widely Expressed Transmitters Involved in Hyperactivity

#### Histamine

Histamine is a widely expressed transmitter signaling—among others—via the Gq-protein coupled H_1_ receptor resulting in a calcium release, involved in the regulation of vomiting, sleep and adrenalin secretion and via the mostly pre-synaptically located H_3_-autoreceptor modulating release of acetylcholine, noradrenalin, and serotonin thus being involved in the regulation of hunger, body temperature, and blood pressure (180).

Male ABA rats exhibited decreased H_1_ receptor binding in the cortex, diencephalon and hippocampus; in contrast, decreased H_3_ receptor binding in cortex and diencephalon due to an acute forced swim test was normalized under conditions of ABA (90). ABA gradually increased central (cortical, diencephalic, and hippocampal) histamine levels and icv administration of additional histamine reduced wheel running activity (90), giving rise to the speculation that the upregulation of central histamine represents a compensatory attempt to reduce hyperactivity. Similarly, humans with AN also showed increased H_1_ receptor binding in the amygdala and lentiform nucleus (181).

Pyrilamine (acute ip injection), an H_1_ receptor antagonist, reduced locomotor activity during the dark period in *ad libitum* fed mice, without exerting effects on food-anticipatory activity under ABA conditions in mice (163).

#### Noradrenaline

Noradrenaline, greatly expressed in the LC, affects various functions including sleep-wake regulation, arousal, attention, and memory (182). ABA in female rats activated LC neurons compared to *ad libitum* fed rats as assessed using the neuronal marker c-Fos (74).

Excessive exercise due to food restriction for 4 days in female Sprague Dawley rats reduced cerebellar noradrenergic fiber length, while exercise in general decreased inter-varicosity interval length and increased varicosity density along noradrenergic fibers (30). Rats that did not respond to the ABA protocol, namely rats that suppressed food restriction-evoked excessive exercise, displayed shortened inter-varicosity intervals resulting in blunting of body weight loss (30). Whether changes in fiber length and varicosity density are cause or result of ABA should be evaluated in further research.

Increased locomotion as a response to food restriction is still a barely understood phenomenon in AN. Therefore, further investigations of the involvement of histamine and noradrenaline might lead to possible strategies to counteract these changes.

### Alterations in Anxiety and Anhedonia and Respective Central Regulatory Areas

ABA in adolescent female mice decreased anxiety as assessed using the elevated plus maze test with a negative correlation between the time in open arms and food restriction-induced wheel activity during the following 24 h; thus, mice displaying high anxiety were hyperactive (183). Using the open field and plus maze tests it was shown that female rats that underwent ABA during adolescence displayed an increased anxiety-like behavior in adulthood, whereas animals subjected to food restriction alone did not (144). The ABA protocol performed twice with 7 days in between in adolescent female rats induced an increased long-term anxiety-like behavior in adulthood as assessed by the elevated plus maze test (97). Animals showed reduced ERbeta signaling in the amygdala using quantitative real-time PCR; however, ovariectomy was unable to prevent long-term behavioral changes (97). Strikingly, another study showed no effect of ABA on anxiety-like behavior as assessed using the elevated plus maze and open field test (49). Refeeding without wheel access after ABA decreased horizontal activity and exploratory horizontal behavior (13).

Male BDNF-Val66Met knock-in mice (BDNF Met/Met) displayed a decreased activity-dependent BDNF secretion and increased anxiety-like behavior (34). Strikingly, under ABA conditions wildtype mice did not differ from BDNF Met/Met mice regarding anxiety and lost GABAergic innervation along distal dendrites in the hippocampal CA1 region and medial prefrontal cortex (34). BDNF Met/Met mice showed reduced food restriction-evoked hyperactivity (34) leading to the hypothesis of blunted vulnerability to ABA.

Only one quarter of female ABA Sprague Dawley rats exhibit transient anhedonia (enduring food restriction and hyperactivity, disappearing during weight restoration) as assessed using the sucrose preference paradigm (94). Noteworthy, exposure to a running wheel correlated with an aversion to sweetened water, and high levels of hyperactivity before food restriction correlated with high susceptibility to body weight loss in ABA (94). Additionally, food-anticipatory activity was related to subsequent food intake only in body weight loss-resistant rats (94). No effect of ABA on unconditioned lick responses to sucrose or quinine or on preference for a diet high in fat could be observed in female rats, indicating no alterations of taste responsivity in ABA rats (49).

Since anxiety and other behavioral changes such as affective, anxiety, obsessive-compulsive, and substance abuse disorders can be observed in AN as well (3), the model also helps to assess these comorbid conditions.

### Alterations in Behavior

When examining operant responding for food reinforcers in male albino ABA rats, each nose-poke response was reinforced by a food pellet during the feeding phase resulting in a steeper decrease in nose-poke response in ABA (84), indicating impaired tolerant learning. ABA also induced an impairment in reversal learning at low weight assessed using the attentional set-shifting test with normalization following weight restoration in female rats (82). ABA in adolescent female rats reduced performance during the novel object recognition task but not in the novel place recognition task or the Barnes maze (49), suggesting impaired contextual but not spatial learning. The underlying central mechanisms warrant further investigation in order to potentially improve treatment of cognitive deficiency in AN.

After experiencing ABA during adolescence until a 25% reduction of baseline body weight and following 10 days of body weight regain, the acquisition of an aversion to sucrose was accelerated and reinforced compared to female control rats (50). Likewise, the extinction process was altered in post-ABA female rats with a significant slowing of extinction in the one bottle test where sucrose is presented for 5 min without following injection (50). These data might provide an underlying mechanism contributing to the high relapse rate in AN (184).

### Alterations of Peripheral Hormones

#### Oxytocin

In food deprived animals, oxytocin within the thymus was decreased, likely due to reduced thymus gland weights also observed under conditions of ABA (86). Similarly, in humans serum oxytocin levels were reported to be decreased in AN compared to healthy controls (185).

#### Corticosteroids

ABA in female Sprague Dawley rats led to higher baseline corticosterone levels (48). Similarly, male and female ABA rats displayed elevated circulating corticosterone concentrations associated with higher relative adrenal gland weights, (86). Interestingly, female rats that developed ABA during adolescence presented increased anxiety-like behavior associated with elevated plasma corticosterone (144). Wheel running induced by hypophagia was absent in adrenalectomized male Lewis rats, a finding reversible by corticosterone replacement (186). Similarly, pre-prandial hyperactivity was diminished by adrenalectomy and restored by acute corticosterone injection (186). Also, in humans with AN, cortisol concentrations were elevated (187), possibly contributing to hyperactivity. The underlying mechanism of corticosterone elevation in AN should be examined in more detail.

#### Ghrelin

Plasma ghrelin levels in female mice were correlated with food-anticipatory behavior observed as running activity under conditions of ABA; conversely, female ghrelin receptor (GHS-R1a) knockout mice did not anticipate food (presented as percentage of total running wheel activity) (81). Similar effects were observed in ABA mice treated either acutely icv or chronically peripherally with a GHS-R_1a_ antagonist that did not show alterations in food intake (81). Additionally, food restriction in ABA C57BL/6 male mice increased preproghrelin mRNA-expressing cells in the stomach proportionally to body weight loss (16). Single daily ip injection of ghrelin and ghrelin combined with IgG from obese, but not lean mice, prevented ABA in male C57BL/6 mice by decreasing physical activity during the feeding period without diminishing body weight loss and altering food-anticipatory activity (18). These inconsistent data, showing decreased running activity under ghrelin receptor knockout conditions as well due to exogenous ghrelin application, could be a result of different routes of application or protocols used. In summary, these results suggest that ghrelin suppression might be an interesting target to tackle hyperactivity in AN.

A study in C57BL/6J and DBA/2J inbred mouse lines showed that food reduction leads to hypoleptinemia. The comparison of both strains additionally showed that C57BL/6J mice reduced wheel activity due to food restriction, while DBA/2J mice displayed hyperactivity correlating with a stronger plasma leptin decline, indicating that dynamic changes of plasma leptin have a greater impact on the development of ABA than a simple reduction of leptin levels (58).

In male Sprague Dawley rats the ABA paradigm was shown to significantly reduce circulating leptin levels and increase ghrelin levels (100). Especially in visceral and gonadal fat leptin was absent (100). Interestingly, expression of ghrelin and leptin (LEPR) receptors was tissue-specifically altered in ABA, with increased GHS-R1a and LEPR expression in oxidative-soleus type of muscle compared to the glycolytic-gastrocnemius type (100). Additionally, GHS-R1a expression in visceral and subcutaneous fat was stimulated by ABA, while the active long form of LEPR was only expressed in subcutaneous fat (100). Disturbed regulation of leptin and ghrelin as seen in ABA rats has also been detected in AN patients (188, 189).

#### Female Reproductive Hormones

Restrictedly-fed female rats with access to wheels losing 25% of body weight in 8 days developed a disruption in the estrous cycle, an alteration restored after weight gain (47). Interestingly, while hypoactivity developing during recovery from ABA disappeared after resumption of estrous cycle, hyperphagia persisted but was limited to nonestrous phases (47). The ABA paradigm repeated twice in adolescent female rats with a 1-week interval in between induced reduced estrogen receptor (ER) beta signaling in the amygdala accompanied by anxiety-like behavior, long-term behavioral changes that could not be prevented by ovariectomy (97). In humans one of the main characteristics of AN is the reduction in estradiol (E2) levels resulting in secondary amenorrhea (190); consequently, this alteration should be mimicked by and further investigated in ABA.

Allopregnanolone, a metabolite of progesterone, sc administered during the 2nd food restriction period had no effect on wheel running activity in mice sensitive to ABA. In contrast, in ABA-resistant female C57BL6 mice it induced increased running activity compared to a resistant group receiving vehicle. Resistance was reflected by a reduction of wheel running in the course of ABA (170).

### Alterations of Glucose Homeostasis and Energy Metabolism

In male ABA rats blood glucose levels and plasma insulin concentrations were decreased compared to *ad libitum* fed or exercising controls; however, peripheral IGF-2 concentrations were elevated (154). Similarly, in male Sprague Dawley rats ABA was shown to significantly reduce fat mass and increase insulin sensitivity (100). Also, female Sprague Dawley rats showed lower insulin levels (48). In male rats subjected to ABA, 2DG led to a reduction of food intake, a finding similarly observed in weight-matched controls suggesting a general effect of weight loss (39). Since hypoglycemia can be a life-threatening consequence in AN (191), the underlying mechanisms should be further examined.

ABA lowered body weight accompanied by an increase in lean/fat mass ratio and fat oxidation in male C57BL/6 mice (13). Refeeding with wheel access after the ABA protocol restored fat free mass (13). In male Wistar rats, wheel running activity reduced malondialdehyde, a degradation product of polyunsaturated fatty acids and a marker of oxidative stress, while food restriction decreased malondialdehyde plasma levels along with antioxidant capacity in liver and catalase activity in the gastrocnemius muscle (91). Additionally, the combination of both, food restriction and access to running wheel, elevated total antioxidant plasma levels but also reduced antioxidant parameters in the liver and plasma malondialdehyde levels compared to controls (91), presumably resulting from a reduced need of antioxidant activity in the liver associated with a higher plasma antioxidant capacity. Additionally, during refeeding after development of ABA, female rats displayed a lower resting energy expenditure and total energy expenditure resulting in higher weight gain, although energy intake was lower compared to controls (95). Although controls and ABA rats maintained similar body weights, lipid accumulation in visceral adipose tissue was reduced, while liver fat accumulation was increased in post-ABA rats, probably caused by overfeeding with carbohydrates (95). Therefore, the ABA model can also be used to study the consequences of weight restoration in AN.

Interestingly, ABA mice exhibited an activation of autophagy as assessed by increased dynamin-1 and LC3II/LC3I ratio (21). This was observed in the soleus muscle of ABA mice associated with reduced protein synthesis, while in the anterior tibialis no alterations were observed. Compared to controls and restrictedly fed mice, C57BL/6 ABA mice displayed a reduction in dihydrolipoyl dehydrogenase and 3-mercaptopyruvate sulfurtransferase and other mitochondrial proteins implicated in energy metabolism (192).

### Gastrointestinal Alterations

Comparing restrictively fed male C57Bl/6J ABA mice with mice on a restricted feeding schedule ABA mice had a thinner muscular layer and decreased claudin-1 expression in the colon associated with increased colonic permeability without differences in occludin expression or jejunal paracellular permeability after 17 days (17). Refeeding after ABA for 5 days without wheel access elevated colonic permeability, indicated by increased FITC-dextran flux compared to levels during ABA. Refeeding with wheel access increased muscle kynurenine conversion into kynurenic acid in male mice. Conversion prevents kynurenine, producing oxygen radicals and neurotoxins, to cross the blood-brain barrier, thus protecting from stress-induced depression, indicating a benefit of physical activity after ABA (13). Female C57Bl/6J ABA mice showed elevated Toll-like receptor 4 (TLR4) mRNA expression on colonic epithelial cells and intestinal macrophages, thus elevating downstream mucosal cytokine production. Simultaneously, hypothalamic interleukin-1β, interleukin-1R_1_, and interleukin-1 receptor-associated kinase 4 as well as plasma corticosterone levels were elevated (19). TLR4-deficient mice displayed a higher mortality rate in response to the ABA protocol (19), suggesting a dual effect of TLR4 in ABA: a key role in the early colonic inflammation and a protective effect, since its absence can be fatal. Noteworthy, ABA in female mice induced an alteration in the colonic mucosal proteome, especially proteins implicated in energy metabolism (192). The mammalian target of rapamycin (mTOR) pathway was decreased inhibiting protein synthesis (puromycin incorporation) and activating autophagy (192), giving rise to an alteration of colonic mucosal proteome due to the downregulation of energy metabolism.

ABA in female rats activated neurons of the NTS [involved in the regulation of gastrointestinal motility (193)] compared to *ad libitum* fed rats as assessed using the neuronal marker c-Fos (74). In female ABA C57BL/6 mice, gastric emptying was delayed compared to controls without food restriction and without wheel access; similarly, animals with limited food access had delayed gastric emptying (20). Food intake microstructure during the feeding period in ABA did not differ from female animals without access to a running wheel during food restriction (74).

In ABA mice, a downregulation of proteins of the antrum was observed, namely ACTA2, VCL, KRT19, KRT8, and DES, proteins implicated in the organization of muscle fiber as well as heat shock proteins STIP1, HSPD1, and HSPA8 (20). Noteworthy, increased levels of gastric oxidized proteins were detected in female ABA mice (20). A total of 52% of rats that achieved a 30% weight loss due to ABA displayed gastric lesions (44) independent of sex but possibly associated with activity stress. Noteworthy, also in patients with AN various gastrointestinal alterations have been described, including decreased gastric emptying, impaired motility, and permeability (194).

### Alterations of the Immune System

Wheel running activity and food restriction increased spleen weight and blastogenic response to lipopolysaccharide in female mice (92). It also decreased serum corticosterone levels, while food restriction alone increased corticosterone levels; however, without a significant correlation between serum corticosterone and any immune system measure (spleen weight and blastogenic response to lipopolysaccharide) (92). Exercise alone had no effect on *in vivo* antibody response to sheep red blood cells and *in vitro* splenic responses to concanavalin A and phytohemagglutinin (92). These observations suggest that exercise might prevent undernutrition-induced immunodepression similar to observations made in AN patients (92).

## Outlook and Conclusion

**Figure 2:**
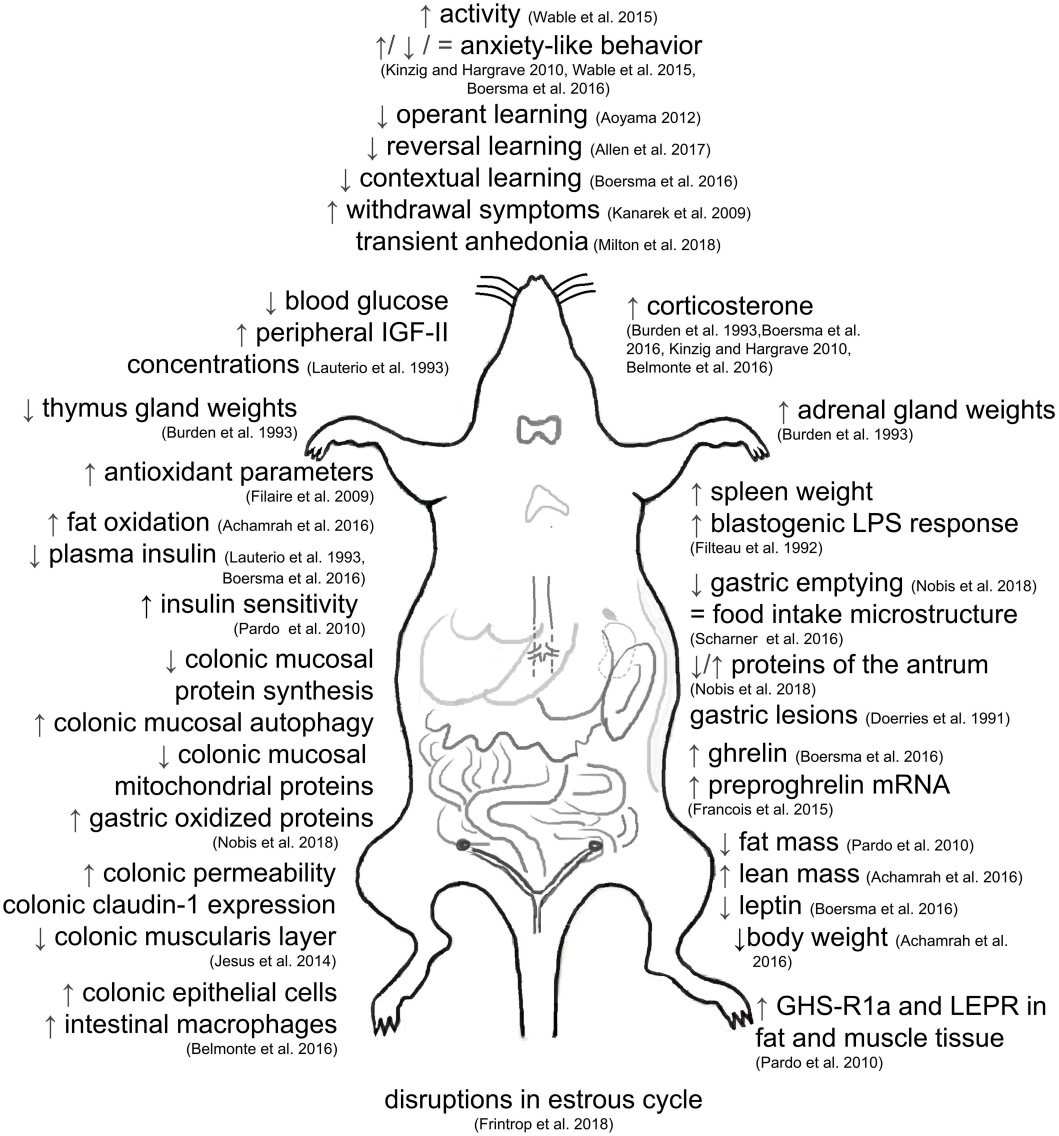
Alterations induced by activity-based anorexia. ↑, increase; ↓, decrease; = , no change.

In summary, ABA induces alterations in different homeostatic systems of the body (Figure 2). In the central nervous system, ABA changes the activity pattern in the motor and higher food intake circuitries as well as the expression of neuropeptides including AgRP, NPY, POMC, and CART in first-order neurons along with NMDA receptor expression. Additionally, hypothalamic protein synthesis is increased. ABA also modulates the GABAergic innervation and integrity of hippocampal structures. The endocannabinoid transmission is affected by ABA as well, along with an increased opioid activity. In addition, abnormal reward signaling can be observed under conditions of ABA. Regarding hormonal alterations, ABA induced an elevation of vasopressin and reduction of oxytocin. Cortisol and ghrelin concentrations are also elevated, while leptin is reduced. Hormonal changes in the reproductive system disrupt the estous cycle in ABA. Hypoglycemia and hypoinsulinemia are accompanied by increased insulin sensitivity in ABA. Functionally, decreased gastric emptying, increased permeability and prevalence of gastric lesions as well as gut inflammation and alterations in the colonic mucosal proteome, especially proteins implicated in energy metabolism, are typical gastrointestinal alterations found in ABA. Noteworthy, the physical activity in ABA can prevent undernutrition-induced immunodepression.

It can be concluded that ABA in rodents shares various similarities with AN in humans. However, as depicted in Tables 1A,B, there is a great heterogeneity of the protocols used to induce activity-based anorexia. Thus, results are often difficult to compare, pointing toward a necessity to standardize the protocol using a specific methodology regarding feeding schedule, acclimatization and housing conditions in order to obtain comparative studies.

To successfully reach the weight loss criterion in ABA, the following methodological details should be regarded: animals with a low body weight should be used, a period of wheel adaptation should be included and food restriction prior to the protocol should be omitted since ABA develops more likely when wheel running interferes with adaptation to the feeding schedule (which is avoided when the feeding schedule is introduced before the running wheel). Additionally, a feeding schedule for <2 h in rats with standard chow and *ad libitum* access to water is necessary to induce hyperactivity and weight loss. Wheel access is crucial especially before food intake. Standard temperature should be guaranteed and isolation should be prevented in ABA, since it extends ABA duration by reducing hyperactivity (54).

Noteworthy, the ABA model has also several limitations. First, ABA only mimics some individuals with AN and not those without hyperactivity, since it combines food restriction and hyperactivity. Although hyperactivity is common in many individuals with AN, it is not present in all cases and it is not a diagnostic criterion. Thus, ABA is no exact replication of the human disease. Second, when animals are placed in conditions with restricted food access and unlimited wheel access as in the present model, there are subgroups of animals who will not engage in running at all; these animals thus preserve body weight and can be maintained on restricted food access for a long time, whereas other animals will fail to consume sufficient food during the period of food access and will run themselves to death in < 1 week (28). Some research groups exclude animals that fail to develop ABA; however, these animals that appear resistant to ABA could serve as a control group. Another limitation is the predominant use of male rodents in the ABA model although the majority of AN patients is female. Lastly, also this systematic review has limitations. Although the data search was performed in three different databases, it cannot be excluded that other relevant publications could not be identified and included. Similarly, a keyword-based search also has flaws, since sometimes keywords are omitted in publications. Moreover, articles in other languages than English were not taken into consideration. Lastly, for reasons of length and readability, not all results of the included publications could be shown.

**Figure 3:**
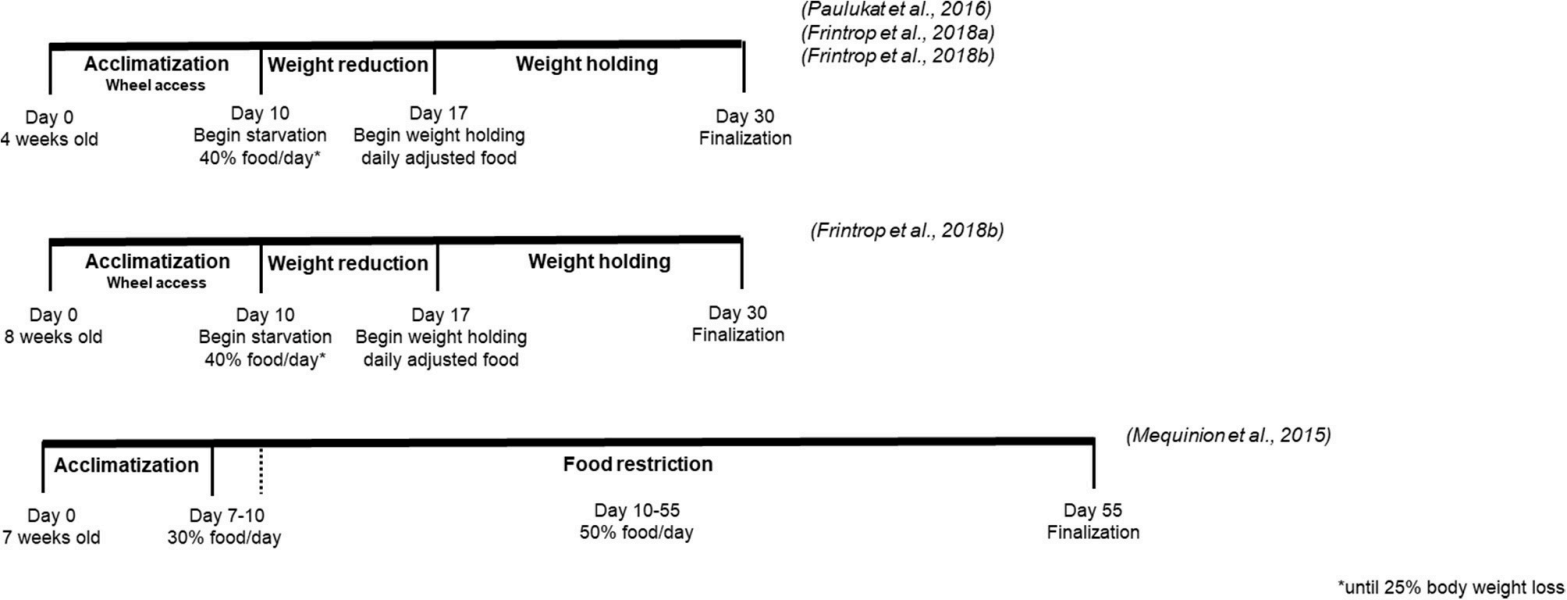
Comparison of different chronic activity-based anorexia protocols.

Since AN is a disease enduring in most cases over several years and has a high relapse rate, in the last years few studies focused on the establishment of a chronic ABA model in order to mimic the pathological alterations in AN more closely (Figure 3). Comparing two protocols, one short-term over 15 days and another over 55 days, in the latter anticipatory hyperactivity was diminished over the course of the protocol, inducing also a reduction of lean mass and body fat as well as reduction of fat oxidation, preferential use of glucose to compensate for the chronic energy imbalance, decrease of leptin levels, increase of corticosterone, and ghrelin concentrations and a disruption of the estrous cycle (14). Wheel access did not prevent loss in bone mineral content due to food restriction and only the long-term protocol induced bone parameters similar to those observed in AN patients (14). Female adolescent rats that lost 20% of body weight due to ABA and subsequently exposed to an additional 2-week weight holding phase displayed a significantly disrupted menstrual cycle and E2 reduction compared to rats whose menstrual cycle was assessed just after the 20% body weight loss (195). Similarly, chronic starvation by food restriction to 40% of the baseline food intake and 24 h/day running wheel access until a 20–25% weight reduction followed by weight holding due to individual food restriction, resulted in a loss of the estrous cycle in all animals with 25% body weight loss, while acute ABA disrupted estrous cycle only in 58% of rats (123). In addition, due to the chronic ABA protocol in female rats an impaired memory function was observed with a correlation between E2 reduction and memory loss (195), possibly giving rise to E2 substitution as therapeutic attempt to treat cognitive deficits in AN. Chronic ABA also reduced the volumes of the cerebral cortex and corpus callosum, Glial fibrillary acidic protein positive astrocytes in these regions, total astrocyte-covered area and astrocyte mRNA expression (196), alterations that likely contribute to the neuropsychological deficits observed in AN. Lastly, comparing different ages, 4-week old rats displayed increased hyperactivity and amenorrhea compared to 8-week old animals (123), indicating that younger animals are more vulnerable to chronic ABA.

However, the chronic ABA models described above used a method of food restriction that includes offering a certain amount of food per day, which is different from the ABA model as stated in the introduction. In the standard model the animal is provided with an unlimited access to food for a limited period of time and has to choose between food intake and running wheel. In the amount-restricted model, animals will typically consume all of the food provided, a difference that should be kept in mind when comparing results. It might be useful to establish a chronic model with time restricted food access. Lastly, only a chronic protocol can induce robust alterations of the menstrual cycle and bone parameter, features also observed in AN. In the chronic protocol, female rodents seem more suited since they endure a longer period without reaching the weight loss criterion (44, 120), while males showed a higher mortality rate due to ABA (13, 15). A chronic model including 4-week old rats losing 25% of body weight seems to most reliably display (chronic) AN characteristics.

## Author Contributions

MS wrote the first draft of the paper. AS thoroughly reviewed the manuscript. Both authors finalized the manuscript.

### Conflict of Interest Statement

The authors declare that the research was conducted in the absence of any commercial or financial relationships that could be construed as a potential conflict of interest.

## Abbreviations

2DG: 2-deoxy-D-glucose
5-HT: serotonin
α-MSH: alpha-melanocyte stimulating hormone
ABA: activtiy-based anorexia
ACTH: adrenocorticotropic hormone
AgRP: Agouti-related protein
AN: anorexia nervosa
ARC: arcuate nucleus
BDNF: Brain-derived neurotrophic factor
BMI: body mass index
CART: cocaine- and amphetamine-regulated transcript
CRF: corticotropin-releasing factor
DMH: dorsomedial hypothalamus
ER: estrogen receptor
GABA, Gamma-aminobutyric acid, GHS-R1a: growth hormone secretagogue receptor 1a
HPA axis: hypothalamus-pituitary-adrenal axis
icv: intracerebroventricular
IGF-2: insulin-like growth factor 2
ip: intraperitoneal
LC: locus coeruleus
LEPR: leptin receptor
LHA: lateral hypothalamus
NAc: nucleus accumbens
NMDA: N-methyl-D-aspartic acid
NPY: neuropeptide Y
NTS: nucleus of the solitary tract
POMC: Pro-opiomelanocortin
PVN: paraventricular nucleus
sc: subcutaneous
SD: Sprague Dawley
SSRI: selective serotonin reuptake inhibitor
TLR4: Toll-like receptor 4
VTA: ventral tegmental area.
